# Iron-Based Nanomaterials/Graphene Composites for Advanced Electrochemical Sensors

**DOI:** 10.3390/nano7120406

**Published:** 2017-11-23

**Authors:** Kaveh Movlaee, Mohmmad Reza Ganjali, Parviz Norouzi, Giovanni Neri

**Affiliations:** 1 Center of Excellence in Electrochemistry, School of Chemistry, College of Science, University of Tehran, 14155-6455 Tehran, Iran; k.movlaee@ut.ac.ir (K.M.); ganjali@khayam.ut.ac.ir (M.R.G.); norouzi@khayam.ut.ac.ir. (P.N.); 2 Department of Engineering, University of Messina, I-98166 Messina, Italy

**Keywords:** iron oxide, hematite, magnetite, maghemite, synthesis, electrochemical sensors

## Abstract

Iron oxide nanostructures (IONs) in combination with graphene or its derivatives—e.g., graphene oxide and reduced graphene oxide—hold great promise toward engineering of efficient nanocomposites for enhancing the performance of advanced devices in many applicative fields. Due to the peculiar electrical and electrocatalytic properties displayed by composite structures in nanoscale dimensions, increasing efforts have been directed in recent years toward tailoring the properties of IONs-graphene based nanocomposites for developing more efficient electrochemical sensors. In the present feature paper, we first reviewed the various routes for synthesizing IONs-graphene nanostructures, highlighting advantages, disadvantages and the key synthesis parameters for each method. Then, a comprehensive discussion is presented in the case of application of IONs-graphene based composites in electrochemical sensors for the determination of various kinds of (bio)chemical substances.

## 1. Introduction

In nanoscale domain, materials often exhibit chemical and physical properties which cannot be observed neither in bulk nor in atom counterparts. Therefore, a great deal of effort has been devoted to prepare nanostructures which offer the desired properties. Thanks to their elegant properties and simplicity of synthesis in laboratory, iron oxide nanostructures (IONs) have achieved a unique position among other nanosized metal oxides [1,2]. Electrical, optical, magnetic and catalytic properties of iron based materials have been exploited for realizing many different purposes in a vast variety of research items. They have been extensively used in supercapacitors, data storage, lithium ion batteries, catalysis, drug delivery, therapeutic agents as well as water treatment [3,4]. Nevertheless, in many cases, it was necessary to combine iron nanostructures with other materials in order to obtain nanocomposites with enhanced performance. In this regard, graphene has shown a great potential to be a valuable option for synthesizing iron oxide/graphene nanocomposites. These hybrid nanostructures have been largely proposed and used for developing advanced devices in many applicative fields. In the chemical sensor field, IONs are exploited extensively as the sensing element part due to their unique electrical and electrocatalytic properties. Among them, electrochemical sensors for the detection of (bio)chemical substances are receiving increasing attention for medical, biological, environmental and industrial applications. Electrochemical sensors provide an attractive means to analyze a variety of analytes in physiological body fluids, food samples, environmental samples and industrial samples, due to the direct conversion of an electrochemical process to an electronic signal. For these characteristics, along with the simple use and low cost, the research for high-performance electrochemical sensors has experienced an explosive growth over the last two decades.

This review will describe iron-based nanomaterials/graphene composites for advanced electrochemical sensors and begin with an introductory part describing the characteristics of the main iron oxide phases used in this regard. Among the different forms of iron oxides [1,2] the most abundant in the nature are hematite (α-Fe_2_O_3_), maghemite (γ-Fe_2_O_3_) and magnetite (Fe_3_O_4_). It is noteworthy that all these forms of iron oxide can also be produced from each other using reducing or oxidizing annealing procedure [2]. As will be seen in the next, magnetite has been largely used in the electrochemical sensor field, however, even if less used, maghemite and hematite show also some interesting applications in this area.

The next section is devoted to acquainting the reader with the most commonly used methods of synthesizing iron oxide nanostructures, along with introducing the advantages, disadvantages and effective parameters of each method. After that, we briefly present information about graphene and, finally, in the last section we will discuss and summarize application of different kinds of composites in which iron nanostructure and graphene have been exploited for the qualitative and quantitative determination of (bio)chemical substances.

## 2. Iron Oxides Nanostructures

Previous published papers related to application of IONs in biochemical detection, deal almost exclusively with hematite, maghemite and magnetite. Therefore, in this review we will mainly focus on them and recommend readers to use other good references for finding extra information about other phases of iron oxides [1,3,4].

### 2.1. Hematite (α-Fe_2_O_3_)

**Figure 1:**
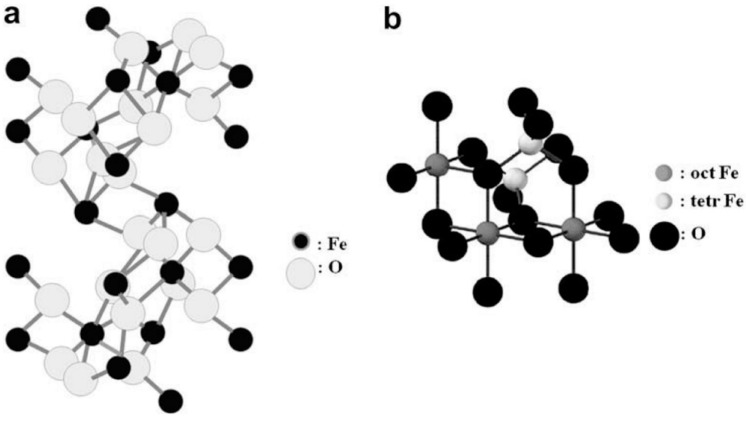
Crystal structures of (**a**) hematite and (**b**) magnetite. Reproduced with permission from [3]. Elsevier, 2009.

Hematite as the oldest known member of iron oxides family is an n-type semiconductor with a band gap of 2.3 eV under ambient conditions which is widespread in rocks and soils and it has shown promising capability in the fields of gas sensors [5,6], pigments [7,8], energy storages [9,10,11,12] and biochemical detections [13,14,15]. This form of iron oxide is highly stable and it is usually the final product of transformation of other iron oxides. If finely divided, hematite has red color and where it is coarsely crystalline shows grey or black color [16]. In hematite structures, empty d-orbitals of Fe^3+^ make conduction band whereas the valence band is made by occupied 3d crystal field orbitals of Fe^3+^ with some admixture from the O 2p non-bonding orbitals [2,17,18]. As shown in Figure 1a, Fe(III) ions fill two-thirds of octahedral positions, which are surrounded with O lattice in almost perfect hexagonal close-packed.

### 2.2. Maghemite (γ-Fe_2_O_3_)

Maghemite is the second most stable form of iron oxides, which is considered as fully oxidized magnetite, see Section 2.3, can be found in natural sources, e.g., soils. Maghemite is weathering product of magnetite to which it is structurally related. Both magnetite and maghemite have a spinel crystal structure, however, while Fe_3_O_4_ contains both di and trivalent iron cations, in maghemite most or all the iron cations are in the form of Fe^3+^ and cation vacancies compensate oxidation of Fe^2+^ [19,20]. In inverse spinel structure of maghemite, Fe(III) ions have been scattered between tetrahedral and octahedral sites. Maghemite shows an n-type semiconductor behavior with a band gap of 2.0 eV [2]. While hematite exhibits antiferromagnetic properties, maghemite is considered as ferromagnetic material which has led to its widespread application in different fields. At about 400 °C, γ-Fe_2_O_3_ is converted irreversibly to α-Fe_2_O_3_ and magnetization will remarkably loss during this conversion [3].

### 2.3. Magnetite (Fe_3_O_4_)

Magnetite, Fe^II^Fe^III^_2_O_4_, is also called iron (II, III) oxide or ferrous ferrite. The molecular formula of magnetite, Fe_3_O_4_, can be shown as (FeO·Fe_2_O_3_). Among all the natural minerals in earth, magnetite possesses the strongest magnetism [21]. Magnetite differs from most other iron oxides since it contains both Fe^2+^ and Fe^3+^ ions. Fe_3_O_4_ has a cubic inverse spinel structure that consists of a cubic close packed array of oxide ions, where tetrahedral site is occupied by Fe^3+^ ions surrounded by four O atoms, while octahedral site is occupied with both Fe^2+^ and Fe^3+^ ions surrounded by six oxygen atoms (Figure 1b). Therefore, Fe^3+^ exists in both tetrahedral and octahedral sites [20]. In magnetite, Fe^2+^ can be fully or partly replaced by other divalent ions such as Co, Mn and Zn. Therefore, magnetite can be both n-type and p-type semiconductor. Owning to its small band gap, 0.1 eV, magnetite has the lowest resistivity among iron oxides [2]. Additionally, fast electron hopping between the Fe^2+^ and Fe^3+^ ions at the octahedral sites brings about high conductivity of Fe_3_O_4_ insofar as magnetite can be considered as half metal [20].

## 3. Preparation Methods of IONs

Since size and shape of nanostructures, size distribution and surface chemistry have great impact on features and behaviors of IONs, preparation methods play a key role in practical aspects. Also, the preparation method determines the degree of structural defects and impurities present in the particles as well as the distribution of such defects thereby manipulation of behaviors of IONs can be achieved. Although IONs can be synthesized in different ways [22], here we introduce just the 4 most commonly used methods which have been extensively exploited for preparation of IONs, i.e., hydrothermal, co-precipitation, microemulsion and sol-gel methods.

### 3.1. Hydrothermal Method

The hydrothermal method is one of the most useful techniques, not only for iron oxide preparation but also for synthesizing other inorganic nanocrystals, especially for metals and metal oxides [20]. Hydrothermal reactions take place in aqueous media in autoclave or reactor where temperature usually needs to be more than 200 °C and the pressure needs to be higher than 13,800 kPa, about 2000 psi, for iron oxide preparation [21].

**Figure 2:**
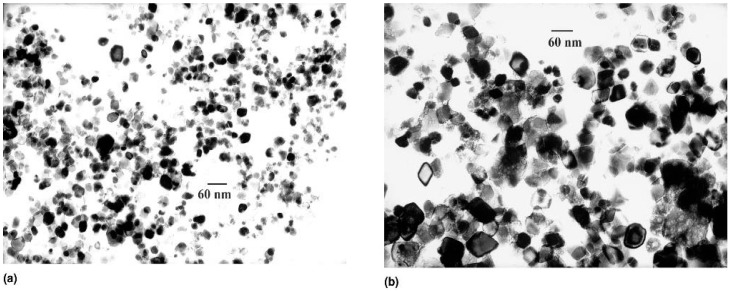
TEM (transmission electron microscopy) pictures of iron oxide nanoparticles obtained at a reactor temperature of 573 K and residence time of 12 s: (**a**) iron feed 0.03 M; (**b**) iron feed 0.50 M. Reproduced with permission from [25]. Cambridge University Press, 2011.

At such high temperature and pressure, metal salts can be undergone hydrolyze and dehydration by water. Consequently, supersaturation is generated due to extremely low solubility of the obtained metal oxides in aqueous media at these elevated pressure and temperature [23]. The elevated temperatures enhance dehydration rates and cause the high diffusivity of reactants in such situations. High supersaturation obtains in this procedure due to the extremely low solubility of metal oxides and hydroxides and finally very fine crystals are prepared [3]. Duo to synergistic effect of high temperatures and pressures the quality of the nanocrystals and hence their magnetic features improve significantly [24]. Some parameters like temperature, pressure, reaction time, type and concentration of precursors can be changed in order to obtain desired shape and/or size of products. Hydrothermal process is environmental friendly since there is no need for using organic solvents or post-treatments. Therefore, hydrothermal technique has been widely used to synthesis metal oxides as powders, nanoparticles and single crystals. Effect of temperature, precursor concentration and reaction time on particle morphology and size in hydrothermal method were investigated by Hao et al. [25]. The particle size and size distribution increased with precursor concentration (see Figure 2). However, the reaction time had stronger effect on the average particle size than feed concentration. Effect of temperature and reaction time can be understood by considering that the formation of the particles occurs in two steps: first nucleation and then crystal growth. At higher temperatures, the nucleation process is faster than the crystal growth, so particles with lower size are obtain; on the other hand, larger particles are produced as a result of longer reaction time where crystal growth becomes determining factor [21,26].

### 3.2. Coprecipitation

Coprecipitation is one of the cheapest, simplest and the most environmental-friendly ways for preparation of IONs which involves the simultaneous precipitation of Fe^2+^ and Fe^3+^ ions in basic aqueous media [24]. A complete precipitation of Fe_3_O_4_ is expected to take place in a media with pH between 9 and 14, while molar ratio of Fe^3+^:Fe^2+^ is 2:1 under a non-oxidizing oxygen-free environment. Fe_3_O_4_ is not very stable, then it can be oxidized into maghemite in the presence of oxygen in solution phase, therefore, in these cases, using of an oxygen free media is necessary to obtain magnetite.

**Figure 3:**
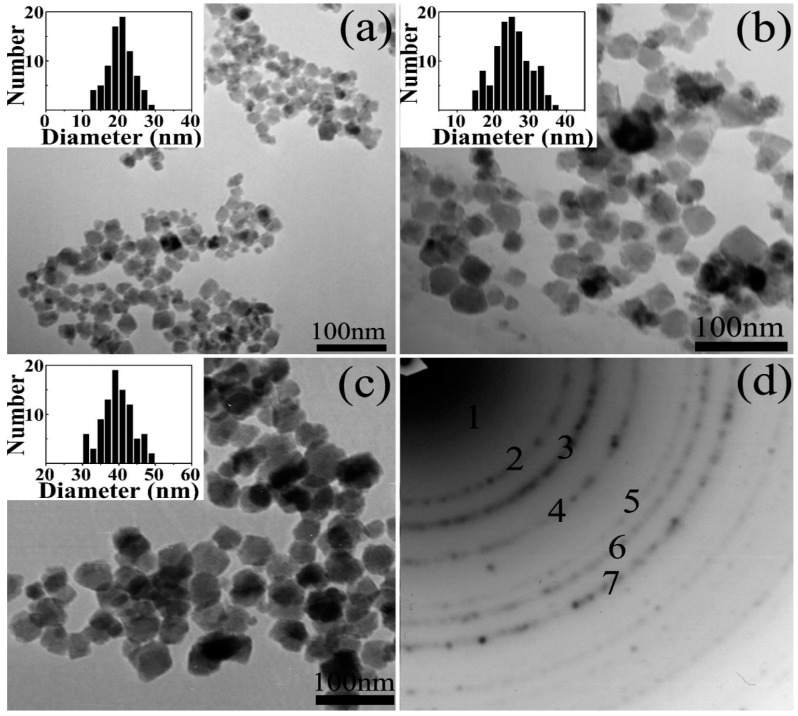
(**a**–**c**) TEM images and size distributions of Fe_3_O_4_ nanoparticles (NPs) with the different mean diameters of 20 nm, (**a**) σ = 16%, 25 nm (**b**) σ = 19% and 40 nm (**c**) σ = 10%. The size distributions show that the synthesized Fe_3_O_4_ NPs had a narrow size distribution. (**d**) Electron diffraction (ED) patterns of the 20-nm Fe_3_O_4_ NPs. Reproduced with permission from [27]. American Chemical Society, 2008.

This method is frequently used in the aqueous phase synthesis of Fe_3_O_4_ nanoparticles. Generally, this method exploits a basic solution, such as sodium hydroxide or ammonia solution to precipitate Fe^2+^ and Fe^3+^. The surface of as-produced IONs is rich of OH groups and these IONs can be easily dispersed in aqueous media [20]. Refait and Olowe proposed a mechanism in which Fe(OH)_2_ can serve as an intermediate for Fe_3_O_4_ formation. The mechanism consists of the precipitation of Fe^2+^ by alkaline, the oxidation of Fe(OH)_2_ by oxygen to FeOOH and the combination of Fe(OH)_2_ and FeOOH to form Fe_3_O_4_. Therefore, they proposed that even if only Fe^2+^ was used as the precursor, Fe_3_O_4_ nanoparticles can be formed using co-precipitation method under air and using Fe^3+^ as precursors is not necessary [20]. Hui et al. prepared large-scale hydrophilic Fe_3_O_4_ nanoparticles and showed possibility of controlling over particle size in the range of 20 to 40 nm by tuning the experimental parameters such as precursor concentration, temperature and ionic strength [27]. Here FeSO_4_·4H_2_O was used as precursor to produce magnetite. As shown in Figure 3, decreasing the ferrous precursor concentration from 0.1 to 0.02 M results in an increase of average size from 20 to 40 nm; for instance, a mean diameter of about 20 nm is obtained by using 0.10 M Fe^2+^ solution. In Figure 3d result of selected area electron diffraction, SAED, for 20-nm Fe_3_O_4_ NPs has been shown which is an illustration for magnetic structure of this sample.

If the concentration of the Fe^2+^ was decreased to 0.05 and 0.02 M, 25 and 40 nm Fe_3_O_4_ nanoparticles would be produced, respectively. This observation can be explained in this way that concentration of Fe^2+^ strongly affects both nucleation and growth rate of Fe_3_O_4_ particles. The higher initial precursor concentration, the smaller particles size due to the formation of a large number of seeds which provide high particle concentration and yield smaller particles.

### 3.3. Sol-Gel

**Figure 4:**
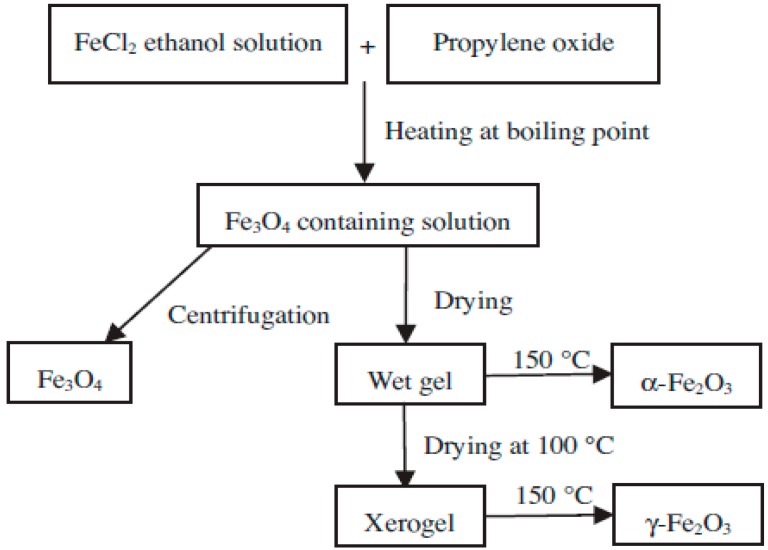
Generalized scheme for the preparation of α-Fe_2_O_3_, γ-Fe_2_O_3_ and Fe_3_O_4_ nanoparticles. Reproduced with permission from [29]. Elsevier, 2013.

This method generally refers to the hydrolysis and condensation of alkoxide precursors and producing a sol which is a dispersion of nanoparticles. Usual precursors for IONs preparation are iron alkoxides and iron salts like chlorides, nitrates and acetates which are undergone various forms of hydrolysis and condensation reactions. Additional condensation and inorganic polymerization causes to form a 3D metal oxide network named as wet gel. Since the reactions are done at ambient, additional heat treatments are required to obtain the final crystalline state [28]. Water is generally used as solvent but precursors can be hydrolyzed by an acid or base. Basic catalysis causes the formation of a colloidal gel whereas acid catalysis results in a polymeric form of the gel [3]. Properties of the final products strongly depend on the rates of hydrolysis and condensation. Smaller particle size is obtained at slower and more controlled hydrolysis rates. Particle size also depends on the solution composition, temperature and pH of solution [2]. It is of great importance to control the rate of hydrolysis and condensation of gel procurers as well as other redox reactions which take place in gelling stage and heat treatments after that since these variables have great impact on structural features and porosity of the final products. Cui et al. [29] synthesized nearly monodispersed Fe_3_O_4_, α-Fe_2_O_3_ and γ-Fe_2_O_3_ nanoparticles using a low temperature sol-gel method with same procedure and same starting materials (see Figure 4)**.** The preparation procedure includes the reaction of FeCl_2_ in boiling ethanol solution with propylene oxide to form a sol solution followed by a drying procedure. Structures of produced IONs easily changed by changing of drying conditions for the sol solution [29].

### 3.4. Microemulsion

A microemulsion is defined as a thermodynamically stable isotropic dispersion of two relatively immiscible liquids that have been stabilized by cationic, anionic and/or non-ionic surfactant like Triton-X. Microemulsions are often clear and stable liquid mixtures of oil, water and surfactant, usually together with a co-surfactant that have been widely used to obtain IONs [2,30,31,32,33,34]. Base on relative concentrations, surfactant molecules self-assemble into different structures in mixture including micelles, bilayers and vesicles. However, micelles are commonly used structures in nanoparticle synthesis, either as normal (oil in water or O/W) or reverse (water in oil or W/O) micelle [35]. In both cases, monodispersed droplets in the size range of 2–100 nm can be produced. This dispersed phase provides a confined environment for synthesizing nanoscale particles. The surfactant-covered water pools produce favorable nano or microenvironments for formation of nanoparticles and, at the same time, for limiting nanoparticles growth. The size of the microemulsion droplets is related to the water to surfactant ratio, although the final size of the nanoparticles may also be changed duo to other parameters like concentration of reactants and flexibility of the surfactant film [3].

**Figure 5:**
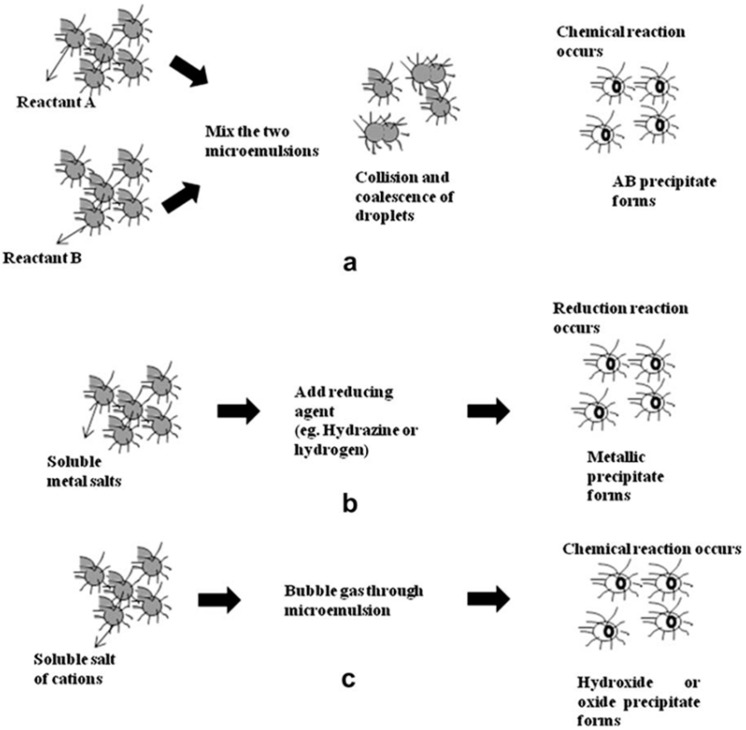
Schematic representation of nanoparticle synthesis in microemulsions (**a**) by mixing two microemulsions; (**b**) by adding a reducing agent; and (**c**) by bubbling gas through the microemulsion. Reproduced with permission from [3]. Elsevier, 2009.

Different ways can be used to produce nanoparticles using microemulsion technique (see Figure 5). For instance, precursors A and B are dissolved in the aqueous phases of two similar W/O microemulsions to form an AB precipitate during mixing. The precipitate is confined to the interior of the droplets thereby size and shape of the particles are determined by droplet size (Figure 5a). In another way, nanoparticles are prepared by adding a precipitating or reducing substance to a microemulsion. This microemulsion consists of the primary reactants which have already been dissolved in an aqueous solution (Figure 5b). These precipitating or reducing substances can be either a gas, e.g., hydrogen, or a liquid, e.g., hydrazine. Figure 5c shows another way to produce carbonate, oxide or hydroxide precipitates. Here microemulsion which contains desired cations is subjected to appropriate bubbling gases such as NH_3_, CO_2_ or O_2_ in order to form IONs [3].

**Figure 6:**
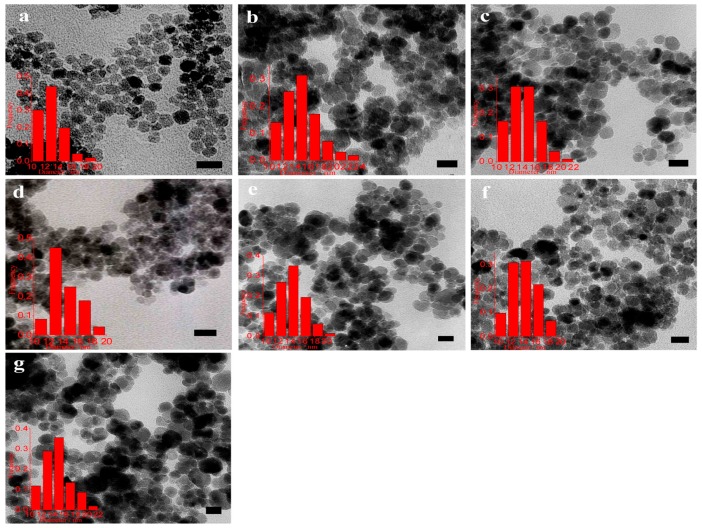
TEM micrographs and size histograms for Fe_3_O_4_ nanoparticles prepared by microemulsion method at 70 °C in (**a**) SDS; (**b**) Brij30; (**c**) DTAB; (**d**) DEAB; (**e**) DBAB; (**f**) CTAB; (**g**) 12-2-12. The scale bar is 20 nm. Reproduced with permission form [34]. Elsevier, 2013.

Lu and coworkers [34] extensively investigated the effect of surfactant on prepared IONs. As shown in Figure 6, different kinds of surfactants can alter the size and/or size distribution of final products. In this study, sodium dodecylsulfate, SDS, polyoxyethylene(4) lauryl ether, Brij30, dodecyltrimethylammonium bromide, DTAB, dodecyltriethylammoniumbromide, DEAB, dodecyltributylammonium bromide, DBAB, and cetyltrimethylammonium bromide, CTAB, were used.

In Table 1, effective parameters that influence on the final product as well as advantage and disadvantages of each method have been summarized.

## 4. Graphene

**Figure 7:**
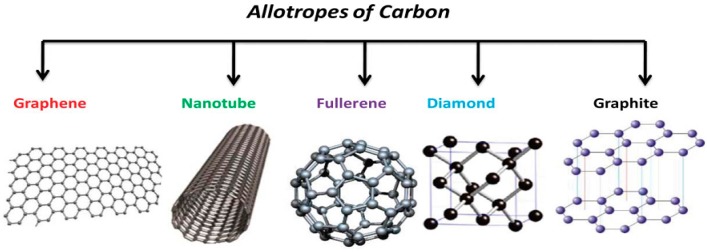
Allotropes of carbon and their crystal structures. Reproduced with permission from [36].

Graphene is a new member of an increasingly populated family of carbon allotropes (Figure 7), which has famous members like graphite, diamond, carbon nanotube, fullerene, graphene, etc. [36].

**Figure 8:**
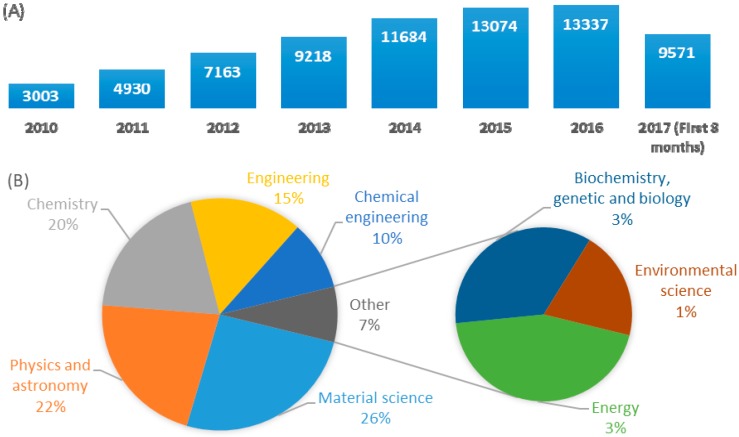
A comparative demonstration of published papers (since 2010 to date) according to Scopus data base searching “graphene” in title. (**A**) published papers since 2010; (**B**) categorized published papers in the first 8 scientific areas according to number of papers.

Graphene has been the center of attention of much scientific research since the early papers of Geim and Novoselov [37], for which they later shared the Nobel Prize in Physics in 2010. Figure 8 clearly shows this increased interest for exploiting of graphene and its derivatives such as graphite oxide, graphene oxide and reduced graphene oxide in scientific papers. Graphene, which refers to a two-dimensional layer of sp^2^ hybridized carbon atoms, exhibits exceptional optical, magnetic, mechanical, electronic and thermal properties as well as large specific surface area [38,39,40]. Due to these extraordinary properties, graphene and its derivatives have been extensively utilized in different areas such as transparent electrode, energy storage, drug delivery, biosensing and catalysis to date [41,42,43,44,45,46,47].

**Figure 9:**
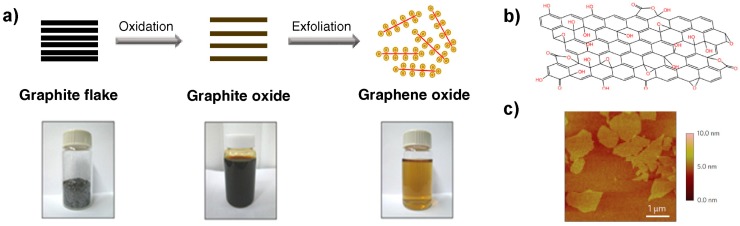
(**a**) Chemical route to synthesize aqueous graphene dispersions; (**b**) The expected chemical structure of a single sheet of graphene oxide (GO); (**c**) AFM (atomic force microscopy) image of the GO on a silicon substrate showing an average thickness of around 1 nm. Reproduced with permission from [45]. Royal Society of Chemistry, 2012.

Graphite oxide possesses similar layered structure to graphite. However, the plane of carbon atoms in graphite oxide is heavily decorated by oxygen-containing groups such as hydroxyl, carboxyl, carbonyl and epoxy groups, which increase the interlayer distance and make these layers more hydrophilic. Therefore, these heavily oxidized layers are easily exfoliated in water under ultrasonication (Figure 9) [48].

Graphene oxide (GO) and reduced graphene oxide (rGO) are other related materials to graphene. GO is layered and oxygenated graphene sheets containing oxygen functional groups and obtains easily as a suspension by exfoliation of graphite oxide. GO can be partially reduced to graphene-like sheets, rGO, by removing the oxygen-containing groups using various reduction methods although some residual oxygen and structural defects will remain even after reduction [48,49].

**Figure 10:**
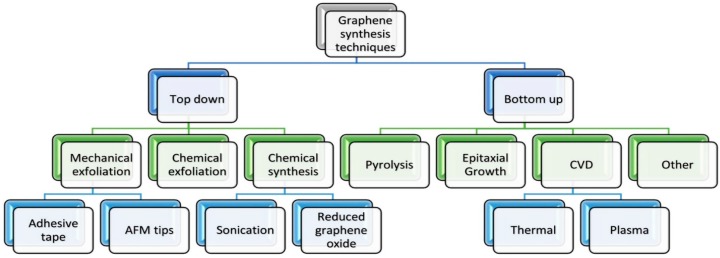
A process flow chart of graphene synthesis. Reprinted with permission from [38].

Synthesis methods of graphene or its related materials can be divided into two general categories namely top-down and bottom-up. Top-down approaches commence with exfoliation of graphite or graphite derivatives such as graphite oxide and graphite fluoride to create the final product e.g., GO or rGO. This approach can be cost effective and scalable depending on the initial materials and yield. In this approach, graphene or rGO are produced by either peeling, cleaving, separation or exfoliation of graphite or its derivatives (Figure 10) [50].

In bottom-up approach, products are synthetized from smaller building blocks (Figure 10). Bottom-up methods encompass standard techniques including epitaxial growth using metallic substrates by means of chemical vapor deposition (CVD) or organic synthesis, which depends on the choice of precursor chemicals and thermal degradation and decomposition of the SiC [38,50].

## 5. Electrochemical Sensors

A chemical sensor can be defined as a device that is able to respond reversibly and continuously to the surrounding environment, providing real-time and reliable information about its chemical composition. These sensors take advantage of a recognition part, which can be a chemical or biological element, joined with a transducing part, which provides an observable signal. Concerning electrochemical sensors, interaction between target(s) and recognition element(s) is converted to an electrical signal by which analytical information can be obtained.

Among different types of transducers that are applied in chemical sensors, e.g., optical, thermal, piezoelectric and so on, electrochemical transducers can offer advantages of low detection limits, wide linear response range, good stability and reproducibility in addition to simplicity, high sensitivity, miniaturization capability and low cost. Owing to these valuable advantages, electrochemical sensors have successfully found their way toward field applications and commercialization. Nowadays many useful electrochemical sensors can be found in the fields of clinical, environmental and industrial analyses [51,52].

**Figure 11:**
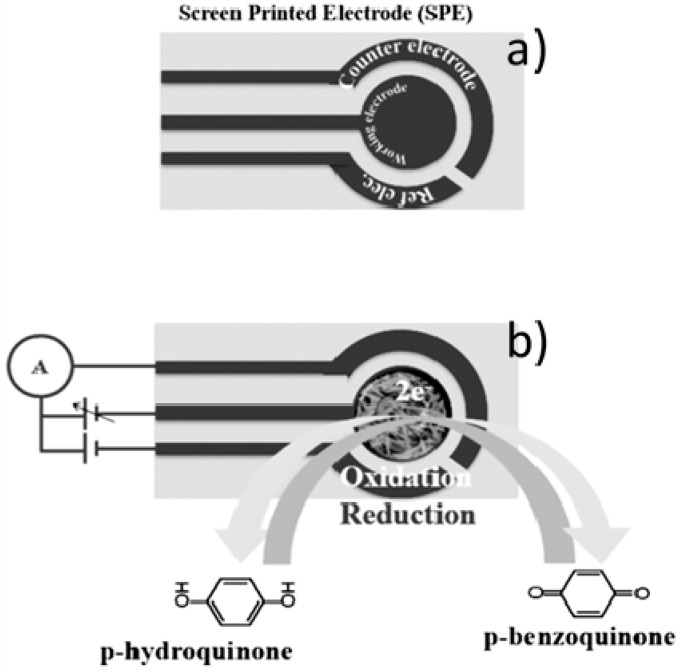
Screen printed electrochemical (SPE) sensor typology. (**a**) conventional SPE; (**b**) modified SPE. Working electrode where the electrochemical reactions happen and the electrical circuit connection are also shown.

Usually, an electrochemical sensor is fabricated assembling a working electrode (e.g., carbon, graphite, platinum, gold), a reference (e.g., Ag/AgCl and calomel) and a counter electrode. In screen printed electrochemical sensor typology (see Figure 11a), electrodes are printed on a ceramic or plastic substrate, making them suitable for mass production, miniaturization and simple use. As described in Figure 11b, working electrode is the zone of the sensor where the recognition layer presents and electrochemical oxidation-reduction reactions happen.

Electrochemical sensors can be classified on the basis of the used recognition element:

*Enzymatic electrochemical sensors*: these sensors are often fabricated by immobilizing an enzyme layer on the surface of the working electrode, which responds to the interactions occurring due to the biocatalytic reactions in the presence of target substances. Enzymes, e.g., phosphatase, peroxidase, etc., are of the most regularly employed biological elements not only in the laboratory experiments but also even in the test kids which have recently found their way to the clinical market. In many cases, especially those exploited in biosensors, interaction of enzymes and targets involves some reduction and/or oxidation reactions, which can be easily converted to an electrical signal using electrochemical transducers. Using enzymes offers such advantages like high selectivity, because enzymes usually bind to their targets in a selective way, as well as high sensitivity and relatively fast response, because enzymes usually show catalytically activity by which sensitivity increases and response time decreases. However, losing activity due to immobilization, relatively high price and showing a loss of activity during a comparatively short time period are some limiting factors for using them.

*Immunosensors*: electrochemical immunoassay methods take advantage of high selectivity of the molecular recognition between the antigen and antibody. Due to its relatively simple device, high sensitivity and ability for miniaturization [53], electrochemical immunoassay has been continuously developed and applied in the field of disease biomarkers detection and other diagnostic tests like pregnancy test. Although immunosensors can be designed by immobilization of either antigens or antibodies, however, immobilization of antigen is more useful owning to this fact that, antibodies are more sensitive biological elements than antigens and immobilization process may lead to loss of affinity as a consequence of structural changing after immobilization [54,55].

*Chemically modified electrochemical sensors*: in these sensors, working electrode is chemically modified deliberately. Generally, electrode modification involves either coating or bounding of desired modifiers (onto electrode surface) that alters electrochemical features of the bare electrodes. Inclusion of electrocatalytic materials within the electrode matrix is another attractive approach for modifying electrodes. Inorganic, organic and hybrid composites can be used for modifying the composition of the electrode to meet specific sensing needs. In addition, these chemically modified electrodes can be used to anchor enzymes, antibodies, aptamers, where the interaction of the effective biorecognition layer with the electrode sensor surface is optimized, ensuring the highest dispersion and a better stability.

As regards the acquisition of the electrical signal, electrochemical sensors fall into three main categories, as follows:

*Potentiometric sensors*: In these kinds of sensors, a potential was established at the surface of recognition part, which is proportional to activity of analyte in a logarithmic fashion. Measured emf at zero current is used as an index for determination of the substance which is being determined. Two kinds of potentiometric sensors i.e., ion selective electrodes and field effect transistors are mostly used for constructing these kinds of sensors. In both of aforementioned potentiometric sensors, a permselective recognition part plays vital role by creating a potential signal that is primarily related to the target ion. Although potentiometric sensors show advantages like simplicity, selectivity, potential capability for multi-elemental analyses in array arrangement and low cost, they usually suffer from less sensitivity and slower response time than their voltammetric counterparts.

*Voltammetric sensors*: these sensors work based on applying a decreasing, increasing or constant potential between working and reference electrodes until reduction or oxidation of analyte(s) occurs and a sharp change in current is appeared. These current changes attributed directly to the concentration of analyte in some conditions. Knowing the oxidation or reduction peak of analyte, one can step the potential to about that value and pursues the current. This method is named amperometry. In all voltammetric methods, electron transfer between electrode and analyte is the key step. Many efforts have been made for modification of electrode surface in order to increase the rate of electron transfer between analyte and electrode and/or hinder this electron transfer for interference species during measurements.

*Electrochemical impedance spectroscopy (EIS)*: this powerful technique has found its way to electrochemical sensors field since last decades and has well proved its ability to provide some valuable information both quantitatively and qualitatively. In this technique, an alternating voltage is often applied and alternating current response will be analyzed with respect to frequency. Two or three electrodes configurations well as frequency response analyzer (FRA) and potentiostat are necessary to perform an EIS measurement [56,57]. Superimposing a constant voltage on alternating voltage is a frequently used strategy during EIS measurements. However, it is necessary to use the lowest possible amplitude for this direct voltage, most often in the range of mV, to avoid of none linearity in the under-study system. According to the apparatus, it is possible to cover a vast range of frequencies, usually from few millihertz to some megaHertz, during EIS and thereby extract some valuable information about under study system [58,59].

## 6. Application

Unique features of IONs e.g., strong magnetic properties, low toxicity, high adsorption ability for immobilization of desired biomolecules and good biocompatibility, together with elegant properties of this new comer member of carbon family e.g., high electrical/thermal conductivity, large surface area and electrocatalytic properties, have stimulated many interests for overcoming difficulties in realizing new scientific ideas or improving the performance of many current devices and methods [60,61,62]. In this review, we will put our particular focus just on application of graphene-IONs in biochemical determination. Catalytic activity of the graphene-IONs can be improved due to enhanced electronic communication e.g., charge transfer between catalyst and support. Additionally, synergistic effects of graphene sheets and IONs components provide nanocomposite with novel physicochemical properties and consequently enhance electrochemical performance. As a result, graphene-IONs nanocomposites have been considered as one of the most promising hybrid materials that can boost the development of more efficient electrochemical sensors [63]. This part is divided into six sections, where each section is related to a single analyte or a group of analytes determined by the graphene-IONs nanocomposites sensors. Table 2 summarizes published papers so far in which graphene-IONs composites are used for the fabrication of the electrochemical sensors.

### 6.1. Glucose

**Figure 12:**
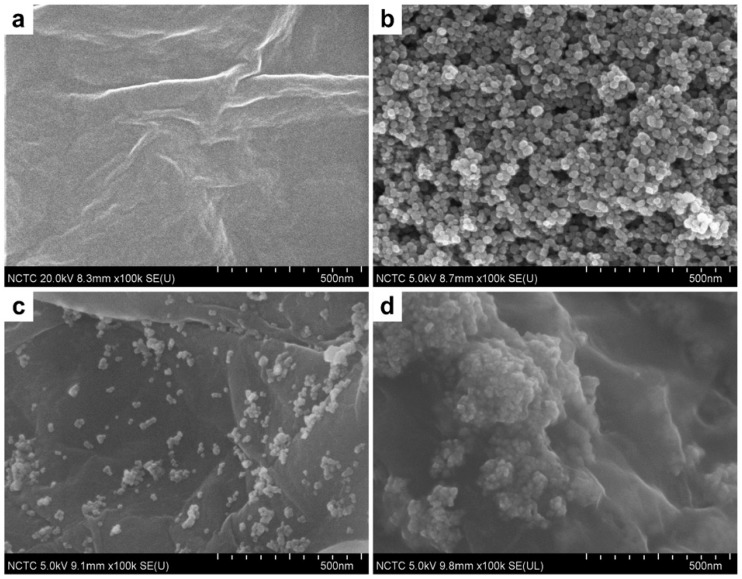
Scanning electron microscopy (SEM) images of (**a**) GO; (**b**) Fe_3_O_4_ NPs; (**c**) RGO-Fe_3_O_4_ nanocomposite and (**d**) RGO-Fe_3_O_4_/Gox nanocomposite. Reproduced with permission from [78]. Elsevier, 2017.

Glucose is a simple sugar that has the molecular formula C_6_H_12_O_6_ and has a vital role in the body. Evaluation of glucose can be useful as a diagnostic test for diabetes mellitus, which is a global health problem with devastating social and economic impact [124] and also for cancer treatment because overexpressed biomarkers on the tumor cells facilitate rates of glucose catabolism and resulting in tumor cell proliferation [125,126,127]. Additionally, determination of glucose level is very important in industrial foods. Pakapongpan and Poo-arporn [78] prepared a rGO-Fe_3_O_4_ nanocomposite (see Figure 12) which exhibited not only remarkable enhancement in surface area for enzyme glucose oxidase (Gox) immobilization but also facilitated electron transfer between enzyme and electrode. Firstly, Fe_3_O_4_ functionalized NH_2_ nanoparticles were synthesized through a solvothermal method. Afterward, NH_2_-Fe_3_O_4_ NPs were bound on GO surface using *N*-Hydroxysuccinimide and 1-Ethyl-3-(3-dimethylaminopropyl)carbodiimide, NHS/EDC, as coupling agents through formation of amide links between the carboxyl group of GO and the amino group of NH_2_-Fe_3_O_4_.

This composite has positive surface charge in neutral pH whereas Gox possesses negative surface charge. This difference in surface charge greatly helps immobilization of Gox on the surface of electrode by electrostatic forces and consequently enhances the performance of biosensor. Interference study showed that common interferences in blood such as ascorbic acid, uric acid and dopamine don not interfere with glucose detection because of high selectivity of Gox. Fast amperometric response, 3 s, wide linear dynamic range of 0.05 to 1 mM and low detection limit of 0.1 μM indicated the abilities of the proposed sensor.

Yang et al. [128] covalently immobilized Gox on the composite of carboxyl long chain modified GO-magnetite nanoparticles using EDC-NHS as binding agent. Although enzyme stability significantly improves by covalent immobilization, activity of enzyme is usually deteriorated due to conformational change of bound enzyme. High selectivity of Gox along with intrinsic hydrogen peroxide catalytic activity of both GO and Fe_3_O_4_ provided this sensor with linear dynamic range of 0.1 to 1.4 mM. Naghib et al. [125] introduced a nonenzymatic glucose sensor based on rGO-Fe_3_O_4_-gelatin composite for determination of glucose in blood sample. This sensor exhibited a wide linear range of 0.1–10 mM and detection limit of 0.024 μM. Improved stability, more than 2 months, was a great advantage of proposed sensor.

### 6.2. Dopamine, Uric and Ascorbic Acid

**Figure 13:**
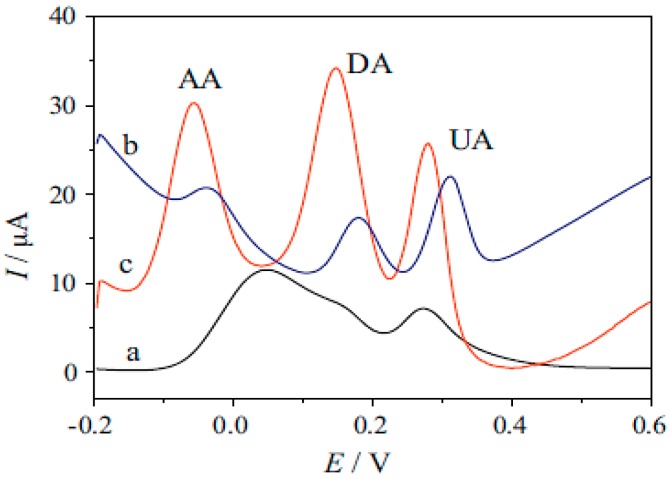
Differential pulse voltammograms at bare GCE (a); GS/GCE (b); Fe_3_O_4_-NH_2_@GS/GCE (c). Reproduced with permission from [130]. Elsevier, 2014.

Dopamine, DA, uric acid, UA and ascorbic acid, AA, coexist in human body and are among the most abundant biomolecules in blood. Therefore, simultaneous determination of DA, UA and AA is very important for the diagnostic research [129]. However, since all of these three biomolecules are easily oxidized, simultaneous determination is often faced difficulty due to overlapping oxidation potentials. Wu et al. [130] resolved this problem by using amino-group functionalized Fe_3_O_4_, Fe_3_O_4_-NH_2_, combined with graphene sheets, GS. Figure 13 shows that three well separated peaks are observed using the modified glassy carbon electrode, Fe_3_O_4_-NH_2_@GS/GCE. Facile solvothermal method was used for preparing of this composite by heating the mixture in an autoclave for 8 h at 200 °C, which was resulted in producing of Fe_3_O_4_-NH_2_ nanostructures anchored GS with average size of 45 nm. Linear dynamic ranges for the determination of AA, DA and UA were 5.0–1600, 0.2–38 and 1.0–850 µmol·L^−1^ under optimum conditions, respectively. Acceptable recoveries in different samples such as DA hydrochloride injection, vitamin C injection and human urine revealed the ability of proposed sensor for application in field.

**Figure 14:**
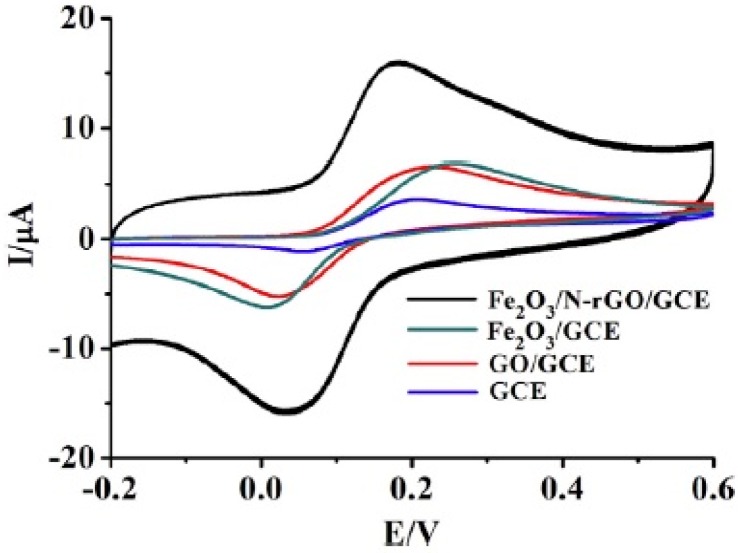
Cyclic voltammograms obtained at GCE, GO/GCE, Fe_2_O_3_/GCE and Fe_2_O_3_/NrGO/GCE in pH 7.5 PBS in the presence of 0.05 mM DA at a scan rate of 100 mV·s^−1^. Reproduced with permission from [13]. Elsevier, 2017.

Fe_2_O_3_-nitrogen-doped reduced graphene oxide, Fe_2_O_3_/N-rGO, was synthesized for enhanced electrochemical determination of dopamine (DA) by Yang et al. [13]. A simple one-step hydrothermal method was used for preparation of nanocomposite in which ethylenediamine served simultaneously as nitrogen source, reducing agent and coordinating agent. Therefore, nitrogen doping and reduction of graphene oxide and producing of Fe_2_O_3_ were occurred in one step. Using this composite for modification of electrode resulted in a higher current (Figure 14). However, charging current had significant share in this current increasing that is not desired for this sensor. Linear range of 0.5 µM–0.34 mM and detection limit of 0.49 µM were reported for this sensor along with capability for determination of DA in dopamine hydrochloride injection as real sample.

Flower like Fe_3_O_4_-reduced graphene oxide nanocomposite [97] and rGO decorated with p-toluenesulfonate-doped polypyrrole/Fe_3_O_4_ nanoparticles [92] were other two composites which were successfully applied for ultrasensitive detection of dopamine with detection limits of 5 nM and 2.33 nM. In an attempt for determination of uric acid, a nanocomposite was prepared by incorporating Fe_3_O_4_ onto graphene sheets followed by depositing of SiO_2_ layer on the surface of the Fe_3_O_4_-rGO composites [79]. This Core-shell Fe_3_O_4_@SiO_2_/rGO nanocomposite was used to modify glassy carbon electrode. It was demonstrated that modified electrode not only increases the peak currents of oxidation of UA but also shifts the oxidation potential of UA to less positive potential. Yu et al. [64] successfully separated overlapped anodic peaks of ascorbic acid and uric acid by exploiting a Fe_2_O_3_-rGO nanocomposite as modifier which had been synthesized by a hydrothermal route. Modified electrode showed synergistic effects by which not only electrochemical oxidation of AA was improved but also overlapping of anodic peaks of AA and UA was resolved. Determination of AA in the presence of UA showed a good linearity from 0.57 to 3.97 mM.

### 6.3. Amino Acids

**Figure 15:**
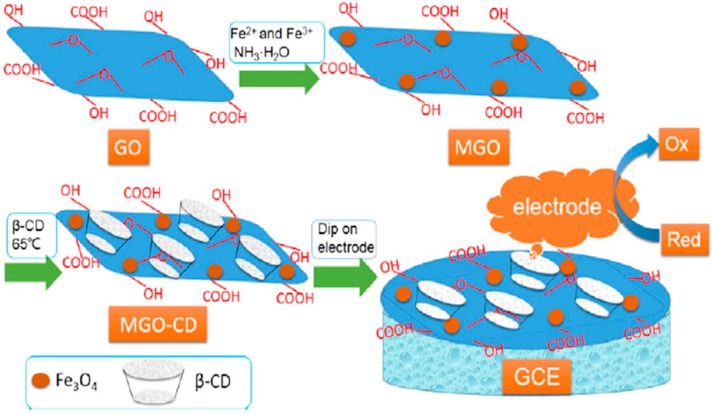
Preparation of β-CD-GO/Fe_3_O_4_ (MGO-CD) nanocomposites and the β-CD-GO/Fe_3_O_4_/GCE (MGO-CD/GCE). Reproduced with permission from [131]. Elsevier, 2016.

Cysteine [67,93], tryptophan [131], phenylalanine [132], tyrosine and aspartic acid [133] are among the most important amino acids which have been successfully quantified using iron based graphene composites as a modifier. A composite of Fe_2_O_3_ nanoparticles supported on N-doped graphene, Fe_2_O_3_NPs/N–GR (Nitrogen doped Graphene), was successfully prepared through a hydrothermal route starting from FeSO_4_·7H_2_O and graphene oxide as reactants followed by dropwise addition of ammonium hydroxide 30% and transferring this mixture to autoclave where it was held for 12 h at 180 °C to obtain abovementioned composite [67]. SEM analysis demonstrated that individual Fe_2_O_3_ NPs with a diameter of about 45 nm have been well spread out on the graphene sheets. Enhanced electrochemical oxidation of cysteine on this modified electrode provided a wide linear determination range of 0.2–400 μM and a lowest detectable concentration of 0.1 μM in 0.1 M phosphate buffer solution pH 7. Wang et al. [93] synthesized a ternary nanocomposite of platinum, magnetite and reduced graphene oxide, referred to as Pt-Fe_3_O_4_/rGO, by a solvothermal method for sensitive determination of cysteine in 0.1 M NaOH. Using this modification allowed them to determined cysteine in the concentration range of 0.1–1 M with a detection limit of 10 µM which was one order of magnitude lower than previous report [67]. Taking advantage of β-cyclodextrin, β-CD, in combination with Fe_3_O_4_ modified graphene oxide (MGO) which had been prepared by a simple coprecipitation method, let Wang et al. [131] to determine tryptophan in the linear range of 5.0 × 10^−7^ to 7.5 × 10^−4^ M with a detection limit of 3.1 × 10^−7^ M. Simple scheme of fabrication of this modified electrode has been shown in Figure 15. The sensitivity was obviously improved owning to the synergistic effects between GO and Fe_3_O_4_, which enhanced the electron transfer rate as well as increased the surface area to capture a large amount of β-CD. Furthermore, β-CD had strong recognition capability for tryptophan due to formation of stable host-guest inclusion complexes leading to the promotion of selectivity.

Hasanzadeh et al. [133] used graphene quantum dots (GQDs) instead of other more common forms of graphene like graphene oxide and reduced graphene oxide in order to prepare a magnetite nanoparticles based composite, Fe_3_O_4_ MNPs-GQDs. In this research, they successfully electrodeposited Fe_3_O_4_ MNPs-GQDs composite on the surface of GCE and exploited that for quantitative determination of l-Cysteine, l-Tyrosine, l-Aspartic acid and l-Phenylalanine. However, although this modified electrode showed ability for analysis of aforementioned amino acids, they reported that determination of l-Serine, l-Arginine, l-Valine and l-Glycine was not possible using this modified electrode.

### 6.4. Deoxyribonucleic Acid

**Figure 16:**
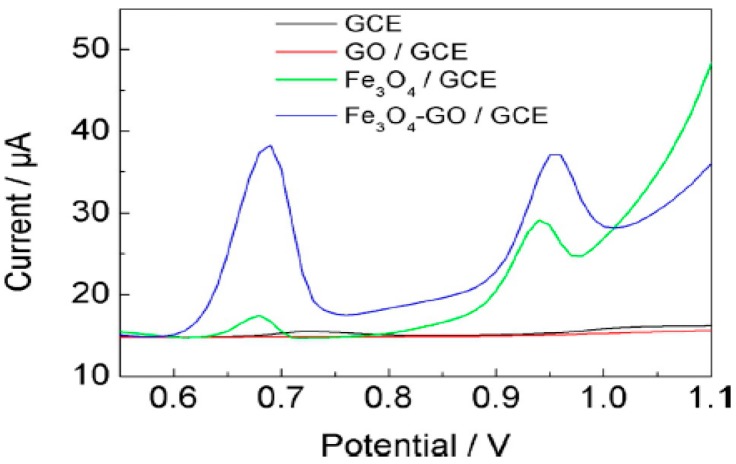
DPVs of bare GCE, GO/GCE, Fe_3_O_4_/GCE and Fe_3_O_4_-GO/GCE in the presence of 20 mM of guanine and adenine in 0.1 M phosphate buffer solution (pH 7). Reproduced with permission from [104]. John Wiley and Sons, 2015.

Detection of specific DNA sequences is attracting growing interest because gene mutations are responsible for inherited human disorders [134]. In addition, pathogens related to viruses and bacteria can be detected by measuring some specific nucleic acid sequences. Guanine and adenine are two important components of DNA which Xie et al. [104] tried to determine them using Fe_3_O_4_-GO composite. Figure 16 clearly shows using Fe_3_O_4_-GO not only increases the peak current but also decreases oxidation potential of guanine and adenine compared to bare GCE and even GCE modified with only GO. This behavior reveals that Fe_3_O_4_ could promote their electron transfer and facilitate oxidation of guanine and adenine.

**Figure 17:**
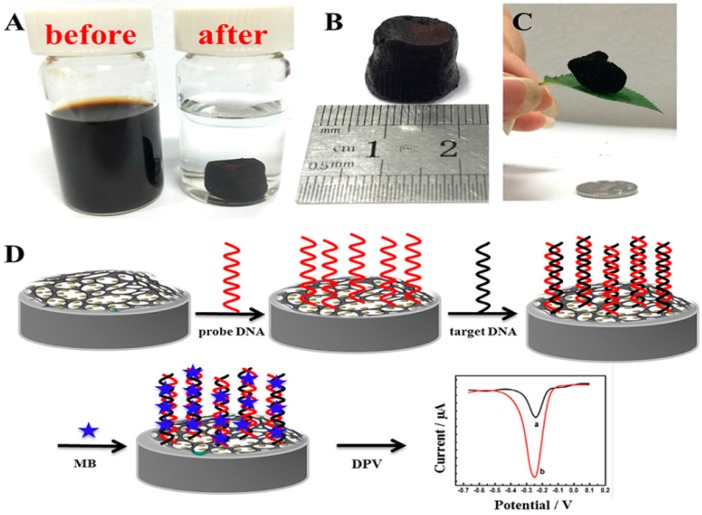
Fabrication process for 3D N-G/Fe_3_O_4_ hydrogels; (**A**) photographs of the mixture of GO, FeCl_3_·6H_2_O and urea in water (before) and reduction and self-assembly of GO to form the 3D gel with embedded Fe_3_O_4_ nanoparticles (after); (**B**) the N-G/Fe_3_O_4_ hydrogel; (**C**) the aerogel of N-G/Fe_3_O_4_ obtained after freeze-drying and thermal treatment; (**D**) Fabrication and detection process of the DNA biosensor. Reproduced with permission from [88]. Elsevier, 2017.

Three-dimensional nitrogen-doped graphene and Fe_3_O_4_, 3D N-G/Fe_3_O_4_, composite was chosen by Chen et al. [88] where highly porous 3D nitrogen-doped graphene provided a large surface area to improve immobilization of probe DNA, whereas magnetite nanoparticles were uniformly spread on the 3D N-G and facilitated electron transfer for sensitive detection of DNA with fast responses. Figure 17 shows some features of 3D N-G/Fe_3_O_4_ composite and simply depicts the procedure of fabrication of this biosensor. Here, during the hydrothermal process for preparation of composite urea served to facilitate the production of Fe_3_O_4_ from Fe^+3^ by altering the basicity of solution and reduction of graphene oxide to N-G simultaneously. This biosensor offered a wide linear range from 1.0 × 10^−6^ to 1.0 × 10^−14^ M and detection limit of 3.63 × 10^−15^ M and ability for detection of target DNA in serum samples using methylene blue, MB, as electrochemical indicator. Jahanbani and Benvidi [135] introduced a magnetic bar carbon paste electrode modified with Fe_3_O_4_NPs-rGO/PANHS nanocomposite in which 1- pyrenebutyric acid-N- hydroxysuccinimide ester ,PANHS, acted as a cheaper linker in comparison to NHS/EDC. Using EIS technique results in linear range of 1.0 × 10^−18^–1.0 × 10^−8^ M and detection limit of 2.8 × 10^−19^ M for this DNA biosensor.

### 6.5. Pesticides

**Figure 18:**
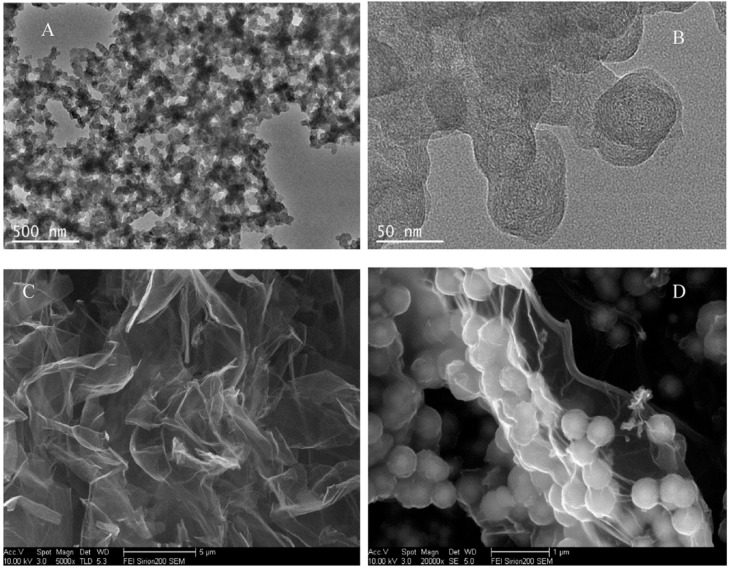
(**A**) TEM image of CB-CS; (**B**) HR-TEM micrographs of CB nanostructure; (**C**) SEM image of GO; (**D**) SEM image of GO@Fe_3_O_4_. Reproduced with permission from [82]. Elsevier, 2017.

Chlorpyrifos (CPF) is one of the most extensively used organophosphorus pesticides in agriculture to control pests and enhances production, whose uncontrolled use causes severe environmental and safety issues. Regarding this, Jiao et al. [82] developed an electrochemical aptasensor for determination of this pesticide in some leafy vegetables, such as cabbage, lettuce and leek. Fe_3_O_4_-GO composite was synthesized by a simple solvothermal method using GO and FeCl_3_·6H_2_O as starting materials and ethylene glycol and ethylene diamine as solvent. Figure 18 shows that Fe_3_O_4_ was successfully integrated with GO and the spherically shaped Fe_3_O_4_ particles with an average diameter of 500 nm. Carbon black and chitosan composite, CB-CS, donated high conductivity to sensor whereas composite of Fe_3_O_4_-GO served with increasing the conductivity and preparing a suitable media for immobilization of aptamer onto the electrode. Linear range of 0.1–105 ng/mL with a low detection limit of 0.033 ng/mL were obtained using this aptasensor for determination of chlorpyrifos.

**Figure 19:**
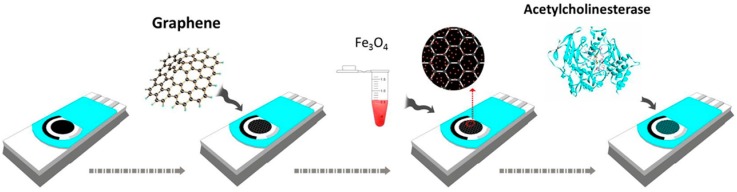
Fabrication and modification process of the sensor. Reprinted with permission from [94].

Wang et al. [94] proposed an enzymatic sensor for determination of chlorpyrifos using acetylcholinesterase (AChE). Figure 19 illustrates fabrication procedure of this sensor. Graphene with high surface area and conductivity along with Fe_3_O_4_ effectively promoted immobilization of AChE and consequently enhanced the performance of biosensor. A linear range from 0.05 μg/L to 100 μg/L and detection limit 0.02 μg/L were reported for this biosensor.

The coprecipitation method was used by Shi et al. [76] for preparation of Fe_3_O_4_-rGO composite in order to determinate trace quantities of omethoate and methamidophos in vegetable samples. Duo to the good dispersion of Fe_3_O_4_ on rGO, the Fe_3_O_4_-rGO had a large surface area and more active sites for electrochemical reactions. Then, it was reported that this sensor has offered rapid responses for these substances in the ranges from 1.0 × 10^−7^ to 1.0 × 10^−12^ mol/L for omethoate and 1.0 × 10^−7^ to 1.0 × 10^−13^ mol/L for methamidophos. Under optimized conditions, the extraordinary low limit of detection of 2.67 × 10^−13^ and 2.05 × 10^−14^ mol/L were reported for methamidophos and omethoate, respectively. Liu et al. [136] reported successfully determination of methyl parathion based on immobilized acetylcholinesterase onto Fe_3_O_4_-rGO nanocomposite film modified glassy carbon electrode with a low detection limit of 3.0 × 10 ^−10^ g·mL^−1^.

### 6.6. Miscellaneous

**Figure 20:**
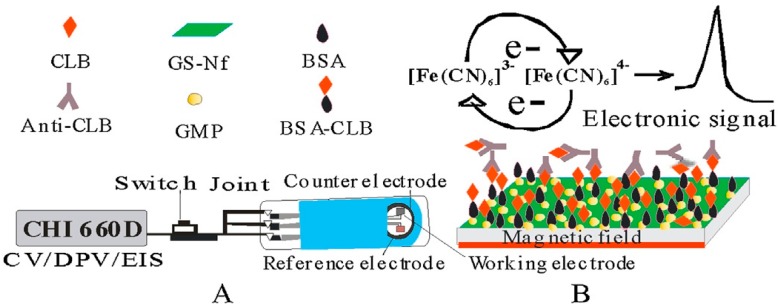
Schematic diagram of the electrochemical immunosensor apparatus (**A**) and the detection principle of CLB with competitive immunoassay mode (**B**). Reproduced with permission from [113]. Elsevier, 2014.

Yang et al. [113] reported the determination of clenbuterol (CLB), a sympathomimetic amine used by people suffering from breathing disorders as a decongestant and bronchodilator, using a magnetic nanocomposite to modify screen printed carbon electrode (SPCE). This immunosensor worked base on competition between free CLB in solution and CLB which had been immobilized on the surface of electrode for binding to CLB-Antibody (see Figure 20). Here, graphene sheets, GN, were mixed with nafion in order to prevent restacking of graphene sheets during chemical reduction of graphene oxide to rGO. Nanocomposite of gold magnetite particle (GMP), was exploited for immobilization of CLB on the surface of SPCE using an external magnetic field under the electrode. Peak current of K_3_[Fe(CN)_6_] probe was used for evaluation of CLB in the samples.

**Figure 21:**
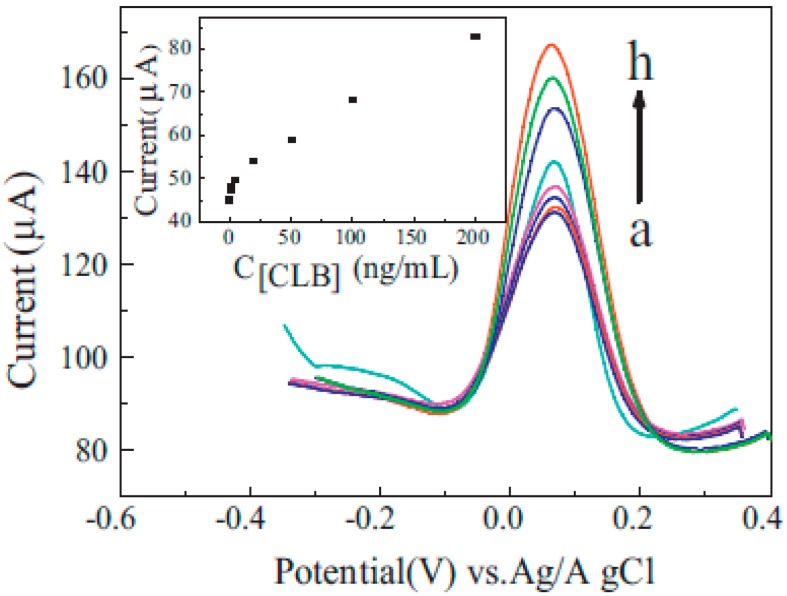
DPVs of the immunosensor incubated in PBS containing 0 (a), 0.5 (b), 2.0 (c), 5.0 (d), 20.0 (e), 50.0 (f), 100.0 (g), 200.0 (h) ng/mL of CLB, 2.0 ng/mL anti-CLB and 2 mmol/L K_3_[Fe(CN)_6_] at 35 °C for 15 min. Inset: The calibration curve of the current values vs. Concentration of CLB. Reproduced with permission from [113]. Elsevier, 2014.

As shown in Figure 21, after using a solution contained K_3_[Fe(CN)_6_], anti-CLB and free CLB, a competition arises between free CLB in solution and immobilized CLB for binding to anti-CLB. The less free CLB in solution, the more anti-CLB binds to CLB on the surface of electrode and, consequently, the more decrease in peak current of probe, K_3_[Fe(CN)_6_], due to steric hindrance of anti-CLB on the surface of electrode.

**Figure 22:**
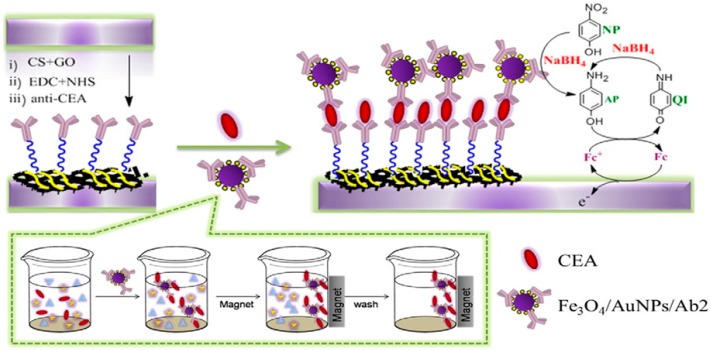
Schematic illustration of the preparation of the immunosensor and the principle of the electrochemical detection. Reproduced with permission from [98]. Elsevier, 2016.

He et al. [117] reported exactly the same strategy for determination of chloramphenicol in milk sample. A sandwich type immunosensor was introduced by Peng et al. [98] for a tumor marker named carcinoembryonic antigen. In this work, Fe_3_O_4_/Au NPs were used for signal amplification, about 10 times and consequently improving the sensitivity of sensor (Figure 22).

Here, graphene oxide was used as the matrix to immobilize large amount of the primary antibodies, AB1, due to the high surface area of GO. Homogeneously distributed Au on Fe_3_O_4_ NPs not only facilitated the assembling of the secondary antibodies, AB2 but also enhanced the electrochemical signal of probe by catalytic effect. Detection limit of 0.39 pg/mL was achieved using this signal amplification method.

Wang et al. [137] used silica nanoparticles loaded with Fe_3_O_4_ nanoparticles and horseradish peroxidase (HRP), as label for a signal amplification strategy. Using this strategy, they determined α-fetoprotein with detection limit of 4 pg/mL thanks to synergistic effect that occurred between Fe_3_O_4_ and HRP for reaction with H_2_O_2_ that greatly enhanced the response signal [138]. This sandwich immunosensor was used successfully for evaluation of α-fetoprotein in serum sample.

Fe_3_O_4_-NH_2_ loaded Pb^2+^ and Cd^2+^ was applied by Zhang et al. [55] as immobilization media for estradiol and diethylstilbestrol antibody in order to simultaneous determination of these two environmental estrogens. Detection limits of 0.015 and 0.38 pg/mL were reported for determination of estradiol and diethylstilbestrol, respectively. PtPd-Fe3O4 nanoparticles (NPs) served as a novel kind of label in a sensitive nonenzymatic sandwich-type immunosensor for detection of gastric cancer biomarker CA72-4 which showed detection limit 0.0003 U/mL [139]. Recoveries between 98.4% and 104% in human serum sample showed the ability of this immunosensor for determination of this cancer marker in real samples. In this sandwich type immunosensor, rGO tetraethylene pentamine (rGO-TEPA) was used for effective immobilization of primary anti-CA72-4 antibody, Ab1. Secondary anti-CA72-4 antibody, Ab2, was adsorbed onto the PtPd-Fe_3_O_4_ NPs.

## 7. Conclusions

IONs-graphene based nanocomposites described in this review offer elegant opportunity for designing high-performance electrochemical sensors which possess the capability to analysis biochemical substances not only in laboratory scale but also in the field application. Valuable electrical and magnetic properties, capability of enhancing electron transfer between electrode and analytes, providing a biocompatible surface for immobilization of biomulecules such as enzymes and DNA, as well as showing electrocatalytic effects in some cases, e.g., for electrochemical reaction of H_2_O_2_, have made IONs-graphene based nanocomposites promising choice in electrochemical sensor application. Nevertheless, innovation in the synthesis of these nanostructures—both in type of ingredients of composite and experimental conditions of synthesis—can still provide different desired properties which are the key point for further enhancements in electrochemical sensors for monitoring of (bio)molecules that is of outmost importance in many applicative fields.

## Figures and Tables

**Table 1:** Different methods for IONs preparation, advantages, disadvantages and effective parameters.

Method	Effective Parameters	Advantages	Disadvantages
Hydrothermal	Pressure temperature reaction time type and concentration of precursors	environmental friendly versatile reactants have elevated reactivity morphology of products can be altered easily producing IONs with good crystallization	need to costly autoclave safety issues usually polydisperse samples are obtained
Sol-gel	rates of condensation and hydrolysis composition of media type and concentration of reactants temperature pH	homogenous and high adhesion products low temperature processing Desired shape and length can obtain	product contains sol–gel matrix components metal alkoxides are almost expensive safety measurements should be considered since large amount of alcohol is released in calcination step
Coprecipitation	concentration of cations counterions pH ionic strength temperature	simplest, cheapest and environmental friendly since the surface of produced IONs using this method contains a great number of hydrophilic ligands, they can be well dispersed in polar solutions, e.g., water	limited by the boiling point of water usually produced iron oxide under these situations shows high polydispersity as well as low crystallinity
Microemulsion	water to surfactant ratio amount of reactants (particularly surfactant) temperature pH surfactant film flexibility	uniform properties narrow pore size distribution	surfactants are difficult to remove difficulty in scale-up procedures

**Table 2:** Application of IONs-graphene composite in biochemical determination.

Iron Oxide	Composite	Analyte(s)	Linear Dynamic Range (LDR)	Limit of Detection (LOD)	Stability	Real Sample(s)	Reference
**Fe_2_O_3_**	Fe_2_O_3_/N-rGO ^1^	Dopamine	0.5 μM to 0.34 mM	0.49 μM	82% of its initial current response after twenty days	Dopamine hydrochloride injection	[13]
	Fe_2_O_3_-rGO	Honokiol	1.5 × 10^−8^~3.3 × 10^−5^ M	9.64 × 10^−9^ M	-	Traditional Chinese medicine	[15]
		Magnolol	7.5 × 10^−8^~2.6 × 10^−5^ M	1.05 × 10^−8^ M			
	Fe_2_O_3_/rGO	AA ^2^	0.57–3.97 mM	0.543 μM	85.3% of the initial currents after 2 weeks	-	[64]
	PEDOT ^3^-rGO-Fe_2_O_3_	Catechol	4 × 10^−8^ to 6.20 × 10^−5^ M	7 × 10^−9^ M	More than 75 days	Green tea	[65]
	NSG-Fe_2_O_3_^4^	Dopamine	0.3–210 μM	0.035 μM	91% of initial current after 7 days	Urine sample	[66]
	Fe_2_O_3_/N-rGO	l-cysteine	0.2–400 μM	0.1 μM	90.6% of initial current after 7 days	Syrup	[67]
	Fe_2_O_3_/rGO	Rutin	1.5 × 10^−8^ to 1.8 × 10^−5^ M	9.8 × 10^−9^ M	-	Tablets	[68]
	PANi ^5^-Fe_2_O_3_-rGO	Hydroquinone	1.0 × 10^−7^–5.5 × 10^−4^ M	6.0 × 10^−8^ M	95.17% of the initial currents after 3 weeks	Tab water	[69]
	GS-Fe_2_O_3_-CTAB ^6^	Bisphenol A	5.0 × 10^−9^–1.0 × 10^−6^ M	2.5 nM	-	Water samples	[70]
	Fe_2_O_3_/rGO	Lysozyme	0.5 ng·mL^−1^–5 μg·mL^−1^	0.16 ng·mL^−1^	95.52% of initial current after 10 days	-	[71]
	Fe_2_O_3_-rGO/CS ^7^	Gallic acid	1.0 × 10^−6^ to 1.0 × 10^−4^ M	1.5 × 10^−7^ M	More than one week	Red and white wines	[72]
**Fe_3_O_4_**	AuNPs/MrGO ^8^	Cortisol	0.1 to 1000 ng/mL	0.05 ng/mL	90.16% of initial current after 20 days	Human serum	[73]
	Fe_3_O_4_-rGO/nafion	Lobetyolin	1.0 × 10^−7^–1.0 × 10^−4^ M	4.3 × 10^−8^ M	93.62% of initial current after 14 days	Radix Codonopsis	[74]
	H-Fe_3_O_4_@C/GNS ^9^	Dopamine	0.1 to 150 μM	0.053 μM	More than 15 days	Rat brain tissue and urine	[75]
		Uric acid	1.0 to 100 μM	0.41 μM			
	(Fe_3_O_4_/rGO) and MIL@MIP ^10^	Methamidophos and Omethoate	1.0 × 10^−7^–1.0 × 10^−12^ M and 1.0×10^−7^–1.0×10^−13^ M	2.67 × 10^−13^ M and 2.05 × 10^−14^ M	94.5% of initial current after 15 days	Cucumber and kidney bean samples	[76]
	Fe_3_O_4_-GO	PSA and PSMA ^11^	1.25–1000 pg/mL and 9.7–5000 pg/mL	1.25 pg/mL and 9.7 pg/mL	80% of initial current after 4 days	Prostate cancer patient serum samples	[77] ^12^
	Fe_3_O_4_-rGO	Glucose	0.05 to 1 mM	0.1 μM	95.6% of initial current after one month	-	[78]
	Fe_3_O_4_-SiO_2_/GO	Uric acid	0.5 to 250.0 μM	0.07 μM	-	Urine sample	[79]
	GS-Fe_3_O_4_/Au@Ag ^13^	CEA ^14^	0.1 pg/mL to 100 ng/mL	0.0697 pg/mL	More than 2 weeks	Human serum samples	[80]
	AuM/N-rGO ^15^	Leukemia cancer cells	10 to 1 × 10^6^ cell mL^−1^	10 cell mL^−1^	-	Human blood plasma	[81]
	CB/Fe_3_O_4_-GO ^16^	Chlorpyrifos	0.1–105 ng/mL	0.033 ng/mL	91.2% of initial current after 20 days	Leafy vegetable	[82]
	Fe_3_O_4_@SiO_2_/GO	Methyldopa (MD)	0.1–400.0 µM	86.0 nM	-	MD tablet and urine samples	[83]
	Fe_3_O_4_-rGO	Sulfonamide	5 × 10^−7^~1.1 × 10^−4^ M	5.0 × 10^−8^ M			[84]
	Fe_3_O_4_-GO/carbon nanotube	Salicylic acid	5.00 to 155 µM	900 nM	-	Water sample	[85]
	magnetic bead-GO/IGZO ^17^	Glucose	3–7 mM	-	-	-	[86]
	Nafion/Mb-SA-Fe_3_O_4_-GR/CILE ^18^	Trichloroacetic acid	1.4 to 119.4 mM	0.174 mM	-	-	[87]
	3D NG-Fe_3_O_4_	DNA	1.0 × 10^−14^ to 1.0 × 10^−6^ M	3.63 × 10^−15^ M	90% of initial current after 2 weeks	Serum samples	[88]
	Fe_3_O_4_-SnO_2_-Gr	AA	0.1 to 23.00 μM	62.0 nM	More than 4 weeks	Biological fluids—pharmaceutical samples	[89]
		DA	0.02 to 2.8 μM	7.1 nM			
		UA	0.015 to 2.40 μM	5.0 nM			
	Fe_3_O_4_@GQD/f-MWCNTs	Progesterone	0.01–0.5 and 0.5–3.0 μM	2.18 nM	85% of initial current after 6 weeks	Serum samples—pharmaceutical products	[90]
	Alginate/Fe_3_O_4_-rGO	Tetracycline	1 nM to 5 μM	0.6 nM	95.86% of initial current after 2 weeks	Food, environmental and clinical samples	[91]
	TSA-doped PPy/Fe_3_O_4_/rGO	Dopamine	7.0–2.0 μM	2.33 nM	More than 10 days	Urine and serum samples	[92]
	Pt-Fe_3_O_4_/rGO	Cysteine	0.10 to 1.0 mM	10 μM	More than 2 weeks	-	[93]
	Fe_3_O_4_-rGO	Chlorpyrifos	0.05 to 100 μg/L	0.02 μg/L	-	Vegetable samples	[94]
	PS/Fe_3_O_4_-GO-SO_3_H ^19^	Doxorubicin	4.3×10^−8^ to 3.5×10^−6^ M	4.9 nM, 14 nM and 4.3 nM	-	Plasma, cerebrospinal fluid, urine	[95]
			8.6×10^−7^ to 13×10^−6^ M				
			2.6×10^−8^ to 3.5×10^−6^ M				
	Pt/Fe_3_O_4_/rGO	NADH ^20^	0.03–1.5 nM	5 nM	-	-	[96]
	Fe_3_O_4_-rGO	Dopamine	0.010 and 0.270 μM	5 nM	93.5% of initial current after 30 days	Urine sample	[97]
	GO/CS-Fc ^21^	CEA ^22^	0.001–30 ng·mL^−1^	0.39 pg	-	Human serum	[98]
	ILFSGo ^23^	Ascorbic acid	1.0 × 10^−6^ to 9.0×10^−4^ M	2.3 × 10^−7^ M	-	-	[99]
	Fe_3_O_4_-rGO	Nitrofuranzone	1.0 × 10^−5^ to 1.09×10^−4^ M	2.92 × 10^−7^ M	-	-	[100]
		Semicarbazide	1.0 × 10^−6^ to 1.09×10^−4^ M	6.17 × 10^−7^ M			
	Fe_3_O_4_-Co_3_O_4_/rGO	Dopamine	5 × 10^−7^ to 1.55×10^−3^ M	1.3 × 10^−7^ M	More than 2 weeks	Human serum samples	[101]
		Uric acid	1.5 × 10^−6^ to 1.6 × 10^−3^ M	1.8 × 10^−7^ M			
	rGO/AuNP/Ab2/S/IMB ^24^	Salmonella pullorum	102 to 106 CFU·mL^−1^	89 CFU·mL^−1^	-	Chicken liver	[102]
	GQD-Fe_3_O_4_/CNT ^25^	L-DOPA	3.0 to 400 μM	14.3 nM	-	Seeds and fava bean	[103]
	Fe_3_O_4_-rGO	Adenine	0.05–25 μM	4 nM	More than 20 days	Fish, urine samples and vitamin B4 tablet	[104]
		Guanine	0.05–25 μM	3 nM			
	Fe_3_O_4_ @ZIF-8 ^26^/RGO	Dopamine	2.0 × 10^−9^ to 1.0 × 10^−5^ M	6.67 × 10^−10^ M	More than 10 days	Urine and serum samples	[105]
	Pd–Fe_3_O_4_-GS	immunoglobulin G	5 × 10^−6^ to 5 ng/mL	3.2 fg/mL	More than one month	Human serum samples	[106]
	Fe_3_O_4_-GO/MIP ^27^	interleukin-8	0.1 to 10 pM	0.04 pM	92.9% of initial current after 1 month	Saliva	[107]
	Fe_3_O_4_-GO@AuNPs-MIP	Dibutyl phthalate	2.5 × 10^−9^ to 5.0 × 10^−6^ M	8×10^−10^ M	96.3% of initial current after 4 weeks	Drink samples	[108]
	AuNPs/Fe_3_O_4_-APTES ^28^-GO	Catechol	2–145 μM	0.8 μM	90% of initial current after 5 days	Tap water	[109]
		Hydroquinone	3–137 μM	1.1 μM			
	MGLA ^29^	APOA2 protein ^30^	0.19 to 1.95 μg·mL^−1^	6.7 pg·mL^−1^	About 80% decrease after one week	Human urine	[110]
	DPSPP ^31^/rGO/Fe_3_O_4_	Hydrazine	120.0–600.0 nM	40.0 nM	-	Water samples	[111]
		Hydroxylamine	10–155.0 μM	3.4 μM			
	rGO/Fe_3_O_4_	Melatonin	0.02–5.80 μM	8.40 × 10^−6^ M	-	Pharmaceutical and biological fluids	[112]
		Dopamine	0.02–5.80 μM	6.50 × 10^−6^ M			
	GS-Nf ^32^/Au-Fe_3_O_4_	Clenbuterol	0.5 ng·mL^−1^ to 200 ng·mL^−1^	0.22 ng/mL	92% of initial current after 4 weeks	Pork sample	[113]
	GS-Au-Fe_3_O_4_	146 antigen (CD146)	5 pg·mL^−1^ to 500 ng·mL^−1^	2.5 pg·mL^−1^	More than 2 months	Human serum samples	[114]
	rGO/Fe_3_O_4_	Ascorbic acid	1–9 mM	0.42 μM	-	-	[115]
		Dopamine	0.5–100 μM	0.12 μM			
	CS-Fe_3_O_4_-GO	Hydroquinone	1.5 to 150 μM	20 nM	95% of initial current after 2 weeks	Tap water	[116]
		Catechol	1 to 410 μM	250 nM			
	GS-Nf/Au-Fe_3_O_4_	chloramphenicol	2.0 ng/mL to 200.0 ng/mL	0.82 ng/mL	93.2% of initial current after one month	Milk sample	[117]
	Fe_3_O_4_-GO-SO_3_H	Furosemide	20–100 μM (serum)	0.1 μM	92.5% of initial current after 20 days	Human serum and urine	[118]
			18–720 μM (Urine)	0.11 μM			
	Iron/nickel oxide nanoparticles-graphene	Orange II	5.0 Nm–3.0 μM	2.0 nM (for all)	-	Different kinds of food samples	[119]
		Allura red and Amaranth	5.0 nM–10.0 μM and 5.0 nM–5.0 μM				
	rGO/Fe_3_O_4_	*N*-acetylcysteine	0.10–10.0 mM	11.1 mM	93.8% of initial current after 2 weeks	-	[120]
	rGO/Fe_3_O_4_	Sudan I	0.008 μM to 6 μM	0.5 nM	-	Food samples	[121]
	Multi-functionalized magnetic graphene sphere	Thyroxine	0.05 pg·mL^−1^ to 5 ng·mL^−1^	15 fg·mL^−1^	85.3% of initial current after 20 days	-	[122]
	Gr-chitosan/Fe_3_O_4_	Guanosine	2.0 × 10^−6^ to 3.5 × 10^−4^ M	7.5 × 10^−7^ M	90.75% of initial current after 15 days	Urine samples and traditional Chinese medicines	[123]

^1^ Fe_2_O_3_/nitrogen-doped Rgo; ^2^ Ascorbic Acid; ^3^ Poly (3,4-ethylenedioxythiophene); ^4^ Nitrogen and sulfur dual doped graphene supported Fe_2_O_3_; ^5^ Polyaniline; ^6^ Hexadecyltrimethylammonium bromide; ^7^ Chitosan; ^8^ Gold nanoparticles-magnetic functionalized reduced graphene oxide; ^9^ Carbon-encapsulated hollow Fe_3_o_4_/GO nanosheets; ^10^ MIL@MIP film as recognition element; ^11^ Prostate specific antigen And prostate specific membrane antigen; ^12^ LDR and LOD were tunable; ^13^ Magnetic graphene loaded gold and silver core-shell nanoparticles; ^14^ Carcinoembryonic antigen; ^15^ Gold-coated magnetic nanoparticles on a nitrogen-doped graphene; ^16^ Carbon black (CB) and graphene oxide@ Fe_3_o_4_; ^17^ Indium gallium zinc oxide; ^18^ Myoglobin (Mb), sodium alginate (SA)-Fe_3_O_4_-GR composite on the carbon ionic liquid electrode; ^19^ Magnetic graphene oxide grafted with chlorosulfonic acid (Fe_3_O_4_-GO-SO_3_H) in the presence of polystyrene; ^20^ Dihydronicotinamide adenine dinucleotide; ^21^ Graphene oxide/chitosan-ferrocene; ^22^ Carcinoembryonic antigen; ^23^ Ionic liquid-magnetic core-shell Fe_3_O_4_@SiO_2_/graphene oxide; ^24^ Immunomagnetic beads; ^25^ Immunomagnetic beads; ^26^ Zeolitic imidazolate framework-; ^27^ Molecularly imprinted polymer; ^28^ (3-Aminopropyl) triethoxysilane; ^29^ Magnetic graphene with longchain acid groups; ^30^ Apolipoprotein A II protein; ^31^ 1-[2,4-Dihydroxy-5-(phenylazo-4-sulphonic acid)phenyl]-1-phenylmethanon; ^32^ Graphene sheets (GS)-Nafion (Nf) film.
